# Comparison of carbapenem MIC for NDM-producing Enterobacterales by different AST methods

**DOI:** 10.1093/jacamr/dlae028

**Published:** 2024-04-06

**Authors:** Alfred Lok Hang Lee, Eddie Chi Man Leung, Viola Chi Ying Chow

**Affiliations:** Department of Microbiology, Prince of Wales Hospital, 30-32 Ngan Shing Street, Shatin, Hong Kong SAR, China; Department of Microbiology, Prince of Wales Hospital, 30-32 Ngan Shing Street, Shatin, Hong Kong SAR, China; Department of Microbiology, Prince of Wales Hospital, 30-32 Ngan Shing Street, Shatin, Hong Kong SAR, China

## Abstract

**Introduction:**

This study compared the performance of MIC test strip (ETEST), automated AST card (Vitek 2) and broth microdilution (BMD) in determining carbapenem susceptibility and MIC values of NDM-producing Enterobacterales.

**Methods:**

NDM-producing Enterobacterales recovered from clinical specimens were included. The presence of *bla*_NDM_ was confirmed by PCR. Identification of bacterial isolates was done by MALDI-TOF. Phenotypic susceptibility to three carbapenems (ertapenem, imipenem and meropenem) was tested by BMD, ETEST and Vitek 2. MIC values were interpreted in accordance with CLSI M100 (2022 edition). Using BMD as the reference standard, the essential agreement (EA), categorical agreement (CA), very major error (VME) and major error (ME) rates were evaluated.

**Results:**

Forty-seven NDM-producing Enterobacterales isolates were included, 44 of which were *Escherichia coli*. The EA of Vitek 2 was 97.9% for ertapenem, 25.5% for meropenem and 42.6% for imipenem. Using Vitek 2, there were 0% VMEs across all three carbapenems tested. The EA of ETEST was 53.2% for ertapenem, 55.3% for imipenem and 36.2% for meropenem. The rates of VMEs for ETEST were high too (ertapenem 8.5%, meropenem 36.2%, imipenem 26.1%). The MIC values obtained from Vitek 2 were consistently higher than those from BMD, while MICs from ETEST were consistently lower than those from BMD.

**Conclusions:**

The VME rate for ETEST was unacceptably high when BMD was used as the standard for comparison. Vitek 2 had acceptable EA and CA for ertapenem when BMD was used as the standard for comparison. For meropenem and imipenem, neither of the methods (ETEST, Vitek 2) showed acceptable EA and CA when compared with BMD.

## Introduction

Carbapenemase-producing Enterobacterales (CPE) are a major threat in antimicrobial resistance. In the Asia-Pacific region, NDM is among the most common types of carbapenemase.^1^ Accurate methods of determining susceptibility of Enterobacterales to carbapenems are required. However, comparison of various antimicrobial susceptibility testing (AST) methods for carbapenems is lacking for this group of common organisms in the literature.

Carbapenem MIC determination is clinically relevant in NDM-producing Enterobacterales (NDM-E). In many parts of the world, newer agents such as cefiderocol or aztreonam/avibactam are of limited availability. In clinical guidelines for management of CPE, carbapenem combination treatment may still be considered for CPE with meropenem MIC ≤ 8 mg/L if newer agents are unavailable.^2^

In this study, we investigated the performance of three common methods for carbapenem MIC for NDM-E isolates. The methods were Epsilometer test (ETEST), automated susceptibility cards (Vitek 2) and broth microdilution (BMD).

## Materials and methods

### Laboratory methods

Forty-seven sequential isolates of NDM-E were collected from non-repeat patients of a university teaching hospital, a district general hospital and a convalescent hospital in 2021 and 2022 in Hong Kong. Laboratory processing was performed in the Department of Microbiology, Prince of Wales Hospital.

Rectal swabs for CPE screening were inoculated onto CHROMID^®^ CARBA SMART chromogenic agar (bioMérieux^®^, Marcy-l’Étoile, France) and incubated at 35°C for 16–24 h under atmospheric conditions. Bacterial colonies with compatible colours were selected for carbapenemase gene PCR (Xpert^®^ Carba-R, Cepheid, CA, USA).

Wound swabs and miscellaneous specimens were inoculated on to blood agar and incubated at 35°C for 16–24 h under 5% CO_2_ conditions. Enterobacterales isolates that tested non-susceptible to either ertapenem or meropenem by disc diffusion were selected for carbapenemase gene PCR.

Identification of organisms was done by MS (MALDI-TOF; Bruker^®^, MA, USA) to species level.

Phenotypic susceptibility to three carbapenems (ertapenem, imipenem and meropenem) was tested by: (i) BMD Sensititre GN7F (Thermo Fisher^®^, MA, USA; Lot number B2034B, expiry date 20 Jan 2024); (ii) ETEST (bioMérieux^®^; ertapenem: Lot number 1007894870, expiry date 29 Jan 2023; Lot number 1008008520, expiry date 26 Mar 2023; Lot number 100910410, expiry date 29 Nov 2024; meropenem: Lot number 1007735350, expiry date 12 Nov 2023; Lot number 1009373600, expiry date 05 Nov 2023; Lot number 1009462280, expiry date 16 May 2023; imipenem: Lot number 1007673600, expiry date 24 Oct 2023; Lot number 1008000470, expiry date 10 Apr 2022; Lot number 1009140460, expiry date 23 Apr 2023; and (iii) Vitek 2 automated AST card AST 258 (bioMérieux^®^; Lot number 7982160203, expiry date 26 Oct 2023).

Inoculum and incubation conditions were followed as per respective manufacturer’s instructions. BMD was performed with reference to CLSI M07 (11th edition, 2018).^3^ The MIC values and the susceptibility categories from each method for each isolate were recorded. Interpretation of carbapenem MIC was performed in accordance with CLSI M100 (32nd edition, 2022).^4^

Control strains used were *Escherichia coli* ATCC 25922, *Klebsiella pneumoniae* ATCC BAA-1705 and *K. pneumoniae* ATCC BAA-1706.

### Statistical analysis

Descriptive statistics were used to summarize the sources and species of bacterial isolates. Using BMD as the standard for comparison, the essential agreement (EA), categorical agreement (CA), very major errors (VMEs) and major errors (MEs) for the three carbapenems (meropenem, ertapenem and imipenem) by the other two methods (Vitek 2, ETEST) were calculated in accordance with CLSI guidance.^5^ EA refers to strains where the evaluated method yielded the same or within one doubling dilution MIC value as the standard for comparison. The acceptable rate for EA was ≥90%. CA refers to strains where the evaluated method yielded an AST result with the same categorical interpretation as the standard for comparison. The acceptable rate for CA was ≥90%. ME refers to strains being interpreted as resistant by the evaluated method, but determined to be susceptible by the standard for comparison. The acceptable rate for ME was <3%. VME refers to strains being interpreted as susceptible by the evaluated method, but determined to be resistant by the standard for comparison. The acceptable rate for VME was <3%.

Comparison of the MIC distribution obtained by different AST methods was done via histograms. The MIC ranges of the three MIC-based methods were different. For a more uniform presentation of graphics, the narrowest ranges of MIC for each antibiotic among the three test methods were chosen for analysis and graphics display.

### Ethics

Ethics committee approval was granted by the Joint Chinese University of Hong Kong—New Territories East Cluster Clinical Research Ethics Committee (project number: 2022.628).

## Results

Among 47 NDM-E isolates, 44 were from rectal swabs and 3 were from wound swabs. Forty-four were *E. coli*, two were *K. pneumoniae* and one was *Proteus mirabilis*.

Table 1 shows the agreement between BMD, Vitek 2 and ETEST. The EA of Vitek 2 was 97.9% for ertapenem, 25.5% for meropenem and 42.6% for imipenem. VMEs of Vitek 2 were 0% across all three carbapenems tested. The EA of ETEST was 53.2% for ertapenem, 55.3% for imipenem and 36.2% for meropenem. VMEs of Etest were high too (ertapenem 8.5%, meropenem 36.2%, imipenem 26.1%). ETEST showed poor CA and EA, and a high VME rate using BMD as reference standard. Vitek 2 showed acceptable VME rates when using BMD as reference standard. VME rates were all lower than 3%. While CA and EA of Vitek 2 for ertapenem were acceptable, this did not hold true for meropenem and imipenem.

**Table 1.** Agreement between BMD, Vitek 2 and Etest

	Ertapenem		Meropenem		Imipenem	
	ETEST	Vitek 2	ETEST	Vitek 2	ETEST	Vitek 2
CA (%)	68.1	100	25.5	76.6	28.3	69.6
EA (%)	53.2	97.9	55.3	25.5	36.2	42.6
VME (%)	8.5	0	36.2	0	26.1	0
ME (%)	0	0	2.2	4.3	0	2.2

BMD was used as the basis of comparison in this table.

Table 2 demonstrates the geometric mean MIC for the three tested carbapenems for each method. The geometric means of ertapenem, meropenem and imipenem MICs were 2.15, 7.26 and 9.00 times higher by Vitek 2 than by ETEST.

**Table 2.** Geometric mean MIC (mg/L) of ETEST, Vitek 2 and BMD for carbapenems

	ETEST	Vitek 2	Sensititre
Ertapenem	2.21	4.76	5.11
Meropenem	1.84	13.36	3.72
Imipenem	1.39	12.51	4.18

**Figure 1.**
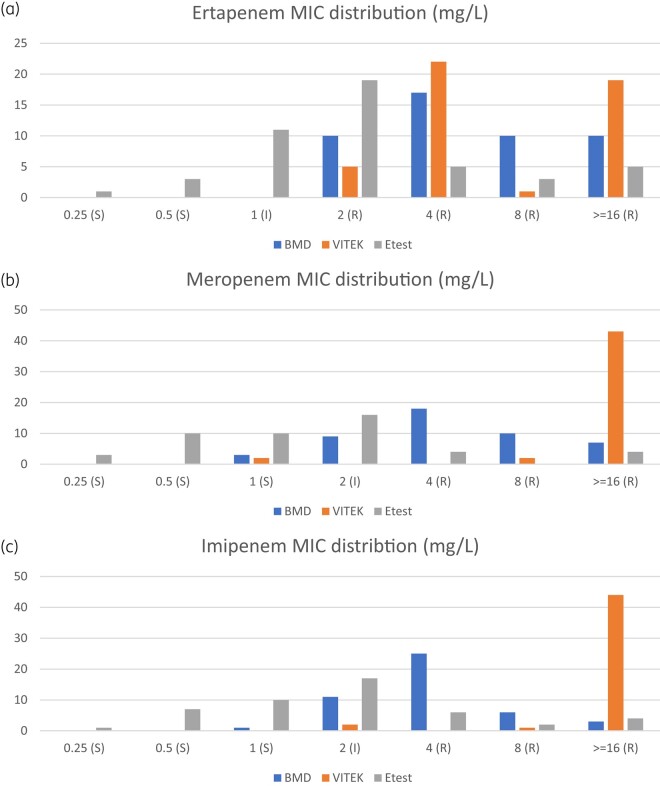
(a) MIC distribution of ertapenem by BMD, Vitek 2 and ETEST. (b) MIC distribution of meropenem by BMD, Vitek 2 and ETEST. (c) MIC distribution of imipenem by BMD, Vitek 2 and ETEST. S, susceptible; I, intermediate; R, resistant.

To further illustrate this difference in MIC, Figure 1(a–c) shows the MIC distribution of ertapenem, meropenem and imipenem based on the three AST methods.

## Discussion

To the authors’ knowledge, this is the first head-to-head comparison study comparing the commonly available AST methods specifically on NDM-E. While BMD is usually regarded as a standard for comparison of AST methods, it is not without drawbacks. It demands significant training of staff, high operating costs, and longer hands-on time than the other two methods. In high-throughput laboratories, the implementation of BMD in determination of carbapenem MIC for CPE requires significant investment. Utilizing the data from this study, the use of Vitek 2 can represent a compromise between accuracy and cost concerns. While the MIC values from BMD and Vitek 2 are not comparable, the susceptibility categorization between the two showed a higher degree of agreement than ETEST.

For the same tested antibiotic agent, the geometric mean MICs derived from different testing methods were significantly different. This shows that while the susceptibility category can be identical, the MIC does not agree between different methods.

Similar issues with ETEST were reported among Enterobacterales producing OXA-48-like carbapenemase.^6^ Out of 82 OXA-48 isolates, ETEST classified 19 (23.1%) as susceptible to ertapenem, compared with 0 isolates by Vitek 2 and BMD. Similar trends were also seen for meropenem and imipenem as well.

There are limitations to this study. The isolates were from a single regional laboratory, and may not represent the entire spectrum of NDM-E globally.

Another limitation is that carbapenemase detection was performed on isolates testing as non-susceptible to carbapenems by disc diffusion or isolates present on screening agars. There are organisms that might have been missed by this approach. A way forward to address this issue would be to perform direct NDM gene PCR for organism selection; however, this poses significant constraints on resources.

In this study, a commercial BMD panel was used instead of MIC-based CLSI reference methods (broth microdilution, agar dilution). While the latter are considered gold standard, they are outside the reach of the usual clinical laboratories. Sensititre would arguably be the closest method a clinical laboratory can access for reference standard MIC determination.

The MIC ranges of various methods are different. For meropenem, all Vitek 2 GN cards have a dilution range of 0.25–16 mg/L. For Sensititre GN7F, the range is 0.5–8 mg/L. In this study, we chose the narrowest range of MIC among the three methods for each antibiotic tested. Comparison of the ‘real’ MIC (e.g. an MIC of 128 instead of ≥16 mg/L for meropenem in GN7F) would be the fairest comparison. Owing to the limitations of testing methods available to most clinical laboratories, this was not possible. However, we would argue this is more reflective of the real-life practice of clinical laboratories, as the test methods evaluated in our studies were all commercially available and commonly used MIC-based test methods.
